# A Selective Fluorescent l-Lactate Biosensor Based on an l-Lactate-Specific Transcription Regulator and Förster Resonance Energy Transfer

**DOI:** 10.3390/bios12121111

**Published:** 2022-12-01

**Authors:** Xianzhi Xu, Rong Xu, Shuang Hou, Zhaoqi Kang, Chuanjuan Lü, Qian Wang, Wen Zhang, Xia Wang, Ping Xu, Chao Gao, Cuiqing Ma

**Affiliations:** 1 State Key Laboratory of Microbial Technology, Shandong University, Qingdao 266237, China; 2 Institute of Medical Sciences, The Second Hospital, Cheeloo College of Medicine, Shandong University, Jinan 250033, China; 3 State Key Laboratory of Microbial Metabolism, School of Life Sciences and Biotechnology, Shanghai Jiao Tong University, Shanghai 200240, China

**Keywords:** l-lactate, biosensor, stereoselectivity, allosteric transcription factor, Förster resonance energy transfer

## Abstract

Selective detection of l-lactate levels in foods, clinical, and bacterial fermentation samples has drawn intensive attention. Many fluorescent biosensors based on non-stereoselective recognition elements have been developed for lactate detection. Herein, the allosteric transcription factor STLldR from *Salmonella enterica serovar Typhimurium* LT2 was identified to be stereo-selectively respond to l-lactate. Then, *ST*LldR was combined with Förster resonance energy transfer (FRET) to construct a fluorescent l-lactate biosensor FILLac. FILLac was further optimized by truncating the N- and C-terminal amino acids of *ST*LldR between cyan and yellow fluorescent proteins. The optimized biosensor FILLac_10N0C_ exhibited a maximum emission ratio change (Δ*R*_max_) of 33.47 ± 1.91%, an apparent dissociation constant (*K_d_*) of 6.33 ± 0.79 μM, and a limit of detection of 0.68 μM. FILLac_10N0C_ was applied in 96-well microplates to detect l-lactate in bacterial fermentation samples and commercial foods such as Jiaosu and yogurt. The quantitation results of FILLac_10N0C_ exhibited good agreement with that of a commercial l-lactate biosensor SBA-40D bioanalyzer. Thus, the biosensor FILLac_10N0C_ compatible with high-throughput detection may be a potential choice for quantitation of l-lactate in different biological samples.

## 1. Introduction

Lactate exists in two stereoisomers: l-lactate and d-lactate [1]. The lactate produced in humans is almost exclusively l-lactate [2]. High l-lactate level indicates serious clinical conditions such as sepsis [3], cardiac arrest [4], and liver failure [5]. l-Lactate level is also an important parameter affecting the flavor or quality of many foods such as wine, dairy products, flavored beverages, and yogurt [6,7]. In addition, l-lactate is a useful chemical with diverse applications. Optically pure l-lactate has been widely used in the synthesis of degradable bioplastic polylactic acid (PLA) [8,9].

Traditionally, the quantification of l-lactate is based on colorimetry [10,11], spectrophotometry [12], fluorescence [13,14], high-performance liquid chromatography (HPLC) [15,16], and liquid chromatography-mass spectrometry [17,18]. However, most of these methods are expensive and involve complicated procedures such as sample pre-treatment and reagent preparation [19]. Biosensors are promising analytical tools which combine a high selective signal recognition element with a signal transducer element. The main advantages of biosensors are their selectivity for a specific analyte and simplicity without tedious sample pre-treatment. Nowadays, many electrochemical biosensors with immobilized enzyme electrodes have been developed for quantification of l-lactate [20,21,22].

Besides electrochemical-based transduction, luminescence-based transduction is also widely applied in biosensors construction [20,21,22,23,24,25]. Laconic, an intracellular biosensor based on the regulator of lactate utilization operon from *Escherichia coli* (*Ec*LldR) and Förster resonance energy transfer (FRET), was first constructed by San Martín et al. to measure the lactate concentration in mammalian cells [26]. Then, many other fluorescent biosensors including GEM-IL [27], Green Lindoblum [28], eLACCO1.1 [29], LARS [30], and LiLac [31], have been developed for quantitative detection of lactate. These biosensors use bacterial allosteric transcription factors (aTFs) [26,27,28], periplasmic binding proteins [29], G-protein-coupled receptors [30], or chemotaxis proteins [31] as recognition elements to detect lactate. However, the recognition elements of the reported fluorescent lactate biosensors are not stereoselective and thus these reported fluorescent biosensors detect both d-lactate and l-lactate. A fluorescent l-lactate biosensor with high stereoselectivity is still urgently needed for quantitative detection of l-lactate.

In this study, the regulator of lactate utilization operon in *Salmonella enterica serovar Typhimurium* LT2 (*ST*LldR) was identified to specifically sense l-lactate. Then, a fluorescent l-lactate biosensor based on this specific aTF and FRET was constructed like Laconic and systematically optimized. The optimal sensor FILLac_10N0C_ was used in specific detection of l-lactate in microbial fermentation samples and commercial foods such as Jiaosu and yogurt.

## 2. Materials and Methods

### 2.1. Materials

d-Lactate, l-lactate, pyruvate, oxaloacetate, citrate, isocitrate, glyoxylate, d-malate, l-malate, succinate, cis-aconitate, l-glutamate, 2-ketoglutarate, l-2-hydroxyglutarate, d-2-hydroxyglutarate, and SYPRO orange dye were purchased from Sigma-Aldrich (St. Louis, MI, USA). Fumarate, Tris and MRS broth were purchased from Beijing Solarbio Biotech Co. Ltd. (Beijing, China). All other chemicals were of analytical grade.

### 2.2. Bacterial Strains and Culture Conditions

Bacterial strains used in this study are listed in Table S1 (Supplementary Material). *E. coli* and its derivative strains were cultured in Luria-Bertani (LB) medium (10 g/L tryptone, 10 g/L NaCl, and 5 g/L yeast extract) at 37 °C with shaking at 180 rpm. *Lactobacillus casei* ATCC 334, *L. plantarum* ATCC 14917, and *L. bulgaricus* ATCC 11842 were grown in MRS medium at 37 °C without agitation. Antibiotics were used at the following concentrations: ampicillin at 100 μg/mL, and kanamycin at 50 μg/mL.

### 2.3. Expression, Purification, and Characterization of Lactate Utilization Operon Regulator LldR

The gene *ST*lldR encoding LldR of *S. Typhimurium* LT2 (*ST*LldR) was amplified from genome of the strain, and then ligated into the plasmid pET28a to construct pET28a-*ST*lldR. The recombinant plasmid pET28a-*ST*lldR was transformed into *E. coli* BL21 (DE3) for *ST*LldR expression. The expression strain was cultured in LB medium at 37 °C until an optical density at 600 nm (OD_600_) of 0.6 and induced with 1 mM isopropyl-β-D-thiogalactoside (IPTG) at 16 °C for 12 h. The cells were harvested and washed twice with buffer A (20 mM sodium phosphate, 500 mM sodium chloride and 20 mM imidazole, pH 7.4), resuspended in buffer A supplemented with 1 mM phenylmethanesulfonyl fluoride (PMSF) and 10% (*v*/*v*) glycerol, and then disrupted by sonication. The cell lysate was centrifuged at 13,000 rpm and 4 °C for 40 min. The supernatant was then loaded onto a 5 mL HisTrap HP column (GE Healthcare, Chicago, IL, USA) preequilibrated with buffer A, and the target protein was eluted with buffer B (20 mM sodium phosphate, 500 mM sodium chloride, and 500 mM imidazole, pH 7.4). The purified *ST*LldR was analyzed by 13% sodium dodecyl sulfate-polyacrylamide gel electrophoresis (SDS-PAGE). The protein concentration was determined using a Bradford protein assay kit (Sangon, Shanghai, China), and stored at –80 °C. The expression and purification of LldR in *E. coli* MG1655 (*Ec*LldR), *Pseudomonas aeruginosa* PAO1 (*Pa*LldR) [32] and *P. fluorescens* A506 (*Pf*LldR) were conducted using the same procedure as above.

### 2.4. Fluorescence-Based Thermal Shift (FTS) Assay

FTS assays were performed using a LightCycler 480 system (Roche, Indianapolis, IN, USA) with the filter set to excitation wavelength 465 nm and emission wavelength 580 nm for SYPRO orange. Each 25 μL reaction mixture contained 10 μM LldR, 5× SYPRO orange, and 1 mM different compounds (l-lactate, d-lactate, pyruvate, oxaloacetate, d-malate, l-malate, citrate, isocitrate, succinate, fumarate, cis-aconitate, acetate, glyoxylate, 2-ketoglutarate, d-2-hydroxyglutarate, l-2-hydroxyglutarate and l-glutamate) in a reaction buffer (20 mM sodium phosphate and 150 mM sodium chloride, pH 7.4). The temperature was increased at a rate of 1.2 °C/s over a temperature range from 25 °C to 95 °C. The melting temperature (T_m_) of LldR was calculated from the negative first derivative value of the raw fluorescence data. Thermal shift temperature (ΔT_m_) was calculated by subtracting the T_m_ of the control from the Tm of LldR with the addition of different compounds. Compounds with ΔT_m_ > 2 °C may be potential ligands of LldR.

### 2.5. Construction and Purification of Fluorescent l-Lactate Biosensor FILLac

The FILLac expression vectors were constructed based on the plasmid pETDuet-*mTFP*-*Venus* carrying *mTFP* gene and *Venus* gene [33]. The original *ST*lldR gene, its truncated variants or variants with artificial linkers were inserted between the *mTFP* gene and *Venus* gene by T5 exonuclease DNA assembly method (TEDA) [34] to construct pETDuet-FILLac_0N0C_ and its variants. The l-lactate biosensor FILLac and its variants were expressed and purified using the same procedure of LldR.

### 2.6. Characterization of FILLac In-Virto

The purified FILLac and different compounds were diluted with 50 mM Tris-HCl buffer (pH 7.4), and then mixed at a volume ratio of 3:1 into a black 96-well microplate for a total detection volume of 100 μL. The final concentration of FILLac in the reaction mixture was 1 μM. The fluorescence intensities of FILLac at 485 nm (mTFP) and 528 nm (Venus) were detected by using an EnSight multifunctional microplate detector (PerkinElmer, USA) with excitation at 430 nm. The dose-response curves of purified FILLac for increasing concentrations of l-lactate (10 nm to 1 mM) were fitted with a [Inhibitor] vs. response-Variable slope (four parameters) models of GraphPad Prism 7.0 as follows:

(1) $$R=R\min+\frac{{\left[L\text{-}Lactate\right]}^{p}{\;\times \;(R}_{max}-R_{min})}{{\left[L\text{-}Lactate\right]}^{p}{+K_{d}}^{p}}\;$$

where *R*, *R*_min_, *R*_max_ refer to the fluorescence emission ratio of Venus to mTFP, the ratio in the absence of l-lactate, and the ratio at saturation concentration of l-lactate, respectively. The [l-Lactate], *K_d_*, and *p* refer to the l-lactate concentration, apparent dissociation constant, and Hill Slope, respectively. The maximum ratio changes (Δ*R*_max_) were calculated according to the following formula:

(2) $$\Delta R_{\max}=\text{−}\frac{R_{max}-R_{min}}{R_{min}}.$$

The linear detection range refers to the l-lactate concentration range corresponding to 10–90% changes in the fluorescence emission ratio (Δ*R*).

The emission spectra of FILLac were recorded at 430 nm excitation with emission from 445 nm to 600 nm in steps of 2 nm. The excitation spectra of FILLac at 380−535 nm were recorded at 550 nm emission, in steps of 2 nm. Effects of pH on FILLac were determined by analyzing the responses of FILLac to l-lactate (0, 1, 10 and 100 μM) diluted with 50 mM Tris-HCl buffer at different pH (4.0, 5.0, 6.0, 7.0, 8.0, 8.5, 9.0, 10.0). Effects of temperature on FILLac were determined by analyzing the dose-response curves for l-lactate at 25, 28, 31, 34, 37, 40, and 45 °C, respectively.

### 2.7. Batch Fermentation for Lactate Production

Three lactate producing strains including *L. casei* ATCC 334, *L. plantarum* ATCC 14917, and *L. bulgaricus* ATCC 11842 were cultured in 100 mL shake flasks containing 50 mL of MRS medium with 1% CaCO_3_ at 37 °C for 24 h, respectively. Then, the fermentation samples were collected and the l-lactate concentrations were determined by HPLC, SBA-40D bioanalyzer, and FILLac, respectively.

### 2.8. Jiaosu and Yogurt Samples Preparation

Three different commercial yogurts (Yogurt A, Yogurt B, Yogurt C) were purchased from a local supermarket. Three different natural fruit and vegetable fermented beverages (Jiaosu A, Jiaosu B, Jiaosu C) were purchased online. The Jiaosu and yogurt samples were diluted with 50 mM Tris-HCl buffer (pH 7.4), and the l-lactate concentrations were determined by HPLC, SBA-40D bioanalyzer, and FILLac, respectively.

### 2.9. Quantification of l-Lactate by HPLC, SBA-40D Bioanalyzer and FILLac_10N0C_

To detect the concentrations of lactate in bacterial fermentation samples, Jiaosu, and yogurt by HPLC, the samples were heated in a metal bath at 105 °C for 15 min, centrifuged at 14,500 rpm for 15 min, then the supernatant was filtered through a 0.22 μm filter. Samples were analyzed using an LC-20AT liquid chromatograph (Shimadzu, Kyoto, Japan) equipped with a RID detector and an Aminex HPX-87H column (300 × 7.8 mm, Bio-Rad, Hercules, CA, USA) at 55 °C. The mobile phase was 10 mM sulfuric acid with a flow rate of 0.4 mL/min. The injection volume was 5 μL, and the total analysis time was 35 min. The stereoisomer composition of lactate in various samples were analyzed using an LC-20AT liquid chromatograph equipped with a UV detector at 254 nm and a chiral column (MCI GEL CRS10W, Tokyo, Japan) at 25 °C. The mobile phase was 2 mM CuSO_4_ with a flow rate of 0.5 mL/min. The injection volume was 5 μL, and the total analysis time was 30 min.

The SBA-40D bioanalyzer (Shandong Academy of Sciences, Jinan, China) was used to quantify the concentrations of l-lactate in various samples. The samples were centrifuged at 12,000 rpm for 2 min, and then the supernatants were appropriately diluted and analyzed for l-lactate concentrations by SBA-40D bioanalyzer.

To evaluate the applicability of FILLac in quantitative analysis of l-lactate in various samples, standard curves of FILLac for l-lactate detection were first established. Purified FILLac_10N0C_ was diluted by 50 mM Tris-HCl buffer (pH 7.4). Increasing concentrations of l-lactate were added to the fermentation medium for detection of fermentation samples, or added to 50 mM Tris-HCl buffer (pH 7.4) for detection of Jiaosu and yogurt. The purified FILLac_10N0C_ and different concentrations of l-lactate were mixed in a black 96-well microplate at a volume ratio of 3:1. After incubating at room temperature for 20 min, the emission intensities were determined at 430 nm excitation by EnSight multifunctional microplate detector. The formula for the quantitative detection of l-lactate concentrations in fermentation samples by FILLac_10N0C_ is as follows:

(3) $$[L\text{-}\mathrm{Lactate}]\;(\mu M)=6.813\;\times {\left(\frac{0.537}{R-1.567}-1\right)}^{0.8347};$$

the formula for the quantitative detection of l-lactate concentrations in Jiaosu by FILLac_10N0C_ is as follows:

(4) $$[L\text{-}\mathrm{Lactate}]\;(\mu M)=6.547\;\times {\left(\frac{0.56}{R-1.696}-1\right)}^{0.9625};$$

the formula for the quantitative detection of l-lactate concentrations in yogurt by FILLac_10N0C_ is as follows:

(5) $$[L\text{-}\mathrm{Lactate}]\;(\mu M)=5.646\;\times {\left(\frac{0.524}{R-1.617}-1\right)}^{0.8576}$$

where *R* refers to the fluorescence emission ratio of Venus to mTFP detected by FILLac_10N0C_. Fermentation samples, Jiaosu, or yogurt were diluted with 50 mM Tris-HCl buffer (pH 7.4), mixed with purified FILLac_10N0C_ and analyzed using the same procedure as mentioned above. The l-lactate concentrations in these samples were determined by substituting the emission ratios into the respective standard curves. Spiking experiments on actual samples were performed by adding 5 mM of l-lactate to three Jiaosu samples, and the concentrations of l-lactate were analyzed using FILLac_10N0C_.

## 3. Results and Discussion

### 3.1. STLldR as a Specific Recognition Element for l-Lactate

aTF contains a DNA-binding domain that binds to specific DNA operator sequences and a ligand-binding domain that senses ligands [35]. Various aTFs have been used as the recognition elements for the construction of fluorescent biosensors [36]. Importantly, some bacterial aTFs can recognize chiral isomers of various metabolites. For example, the transcriptional regulators LhgR from *P. putida* W619 and DhdR from *Achromobacter denitrificans* NBRC 15125 can recognize the l-enantiomer and d-enantiomer of 2-hydroxyglutarate, respectively [33,37]. LhgR and DhdR have been used as the recognition elements to develop fluorescent biosensors for l-2-hydroxyglutarate and d-2-hydroxyglutarate, respectively. Lactate utilization in microorganisms is negatively regulated by transcriptional regulator LldR [38]. To identify the specific transcription regulator for l-lactate, the lactate utilization operon regulator LldRs in *E. coli* MG1655 (*Ec*LldR), *P. aeruginosa* PAO1 (*Pa*LldR), *P. fluorescens* A506 (*Pf*LldR), and *S. Typhimurium* LT2 (*ST*LldR) were overexpressed and purified, respectively (Figure S1, Supplementary Material). Then, the specificities of these LldRs were analyzed by FTS assays.

**Figure 1:**
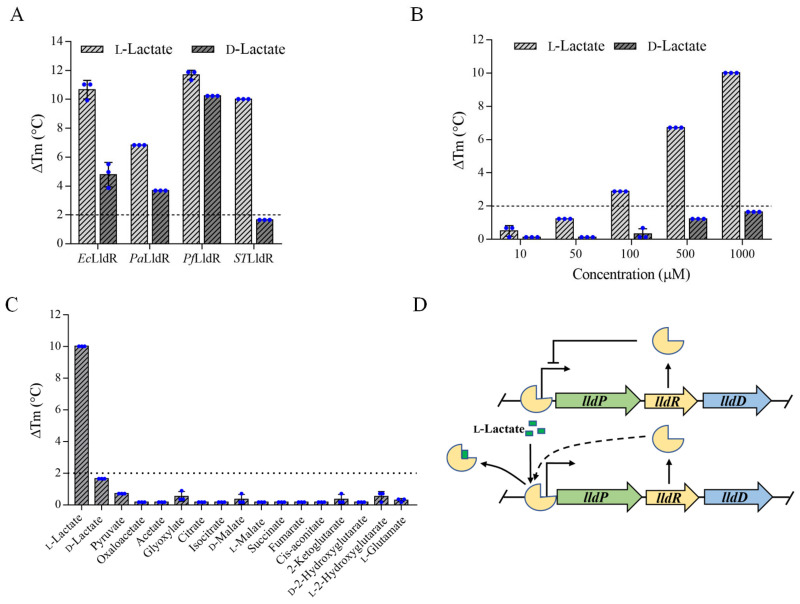
Identification of the l-lactate specific recognition element for biosensor construction. (**A**) Analysis of the interactions of *Ec*LldR, *Pa*LldR, *Pf*LldR, and *ST*LldR with l-lactate and d-lactate by FTS assays. The ΔT_m_ values refer to the changes in the T_m_ of different LldRs in absence or presence of 1 mM d-lactate or l-lactate. (**B**) Comparison of the interaction of *ST*LldR with increasing concentrations (10, 50, 100, 500, and 1000 μM) of l-lactate and d-lactate. (**C**) Specificity analysis of *ST*LldR. The ΔT_m_ values of *ST*LldR were measured in the presence of 1 mM l-lactate, d-lactate, pyruvate, oxaloacetate, acetate, glyoxylate, citrate, isocitrate, d-malate, l-malate, succinate, fumarate, cis-aconitate, 2-ketoglutarate, d-2-hydroxyglutarate, l-2-hydroxyglutarate, l-glutamate. (**D**) Schematic of the regulatory mechanism of the l-lactate utilization in *S. Typhimurium* LT2. *ST*LldR represses the expression of *lldPRD* genes. l-Lactate serves as the effector of *ST*LldR and prevents *ST*LldR binding to the promoter region of *lldPRD* operon. The direction of gene translation in the *lldPRD* operon is indicated by the arrow. LldP, lactate permease; LldR, transcription regulator; LldD, l-lactate dehydrogenase. All data shown are mean ± standard deviations. (s.d.) (*n* = 3 independent experiments).

As shown in Figure 1A, both d-lactate and l-lactate induced significant changes in T_m_ of *Ec*LldR, *Pa*LldR, and *Pf*LldR, while l-lactate but not d-lactate caused a significant change in T_m_ of *ST*LldR. When the concentration of d-lactate was increased to 1 mM, the thermal stability of *ST*LldR was still not significantly changed, while 100 μM l-lactate caused a significant change in thermal stability of *ST*LldR (Figure 1B). In addition, among the seventeen metabolites (1 mM) tested, only l-lactate could lead to a significant change in the thermal stability of *ST*LldR (Figure 1C). The lactate utilization operon of *S. Typhimurium* LT2 is composed of the lactate permease-encoding gene *lldP*, the transcriptional regulator LldR-encoding gene *lldR*, and the l-lactate dehydrogenase-encoding gene *lldD* [39]. Based on the results of FTS assays, we propose that *ST*LldR negatively regulates the l-lactate catabolism of *S. Typhimurium* LT2, and l-lactate is its specific effector (Figure 1D).

### 3.2. Design and Optimization of the Fluorescent l-Lactate Biosensor

**Figure 2:**
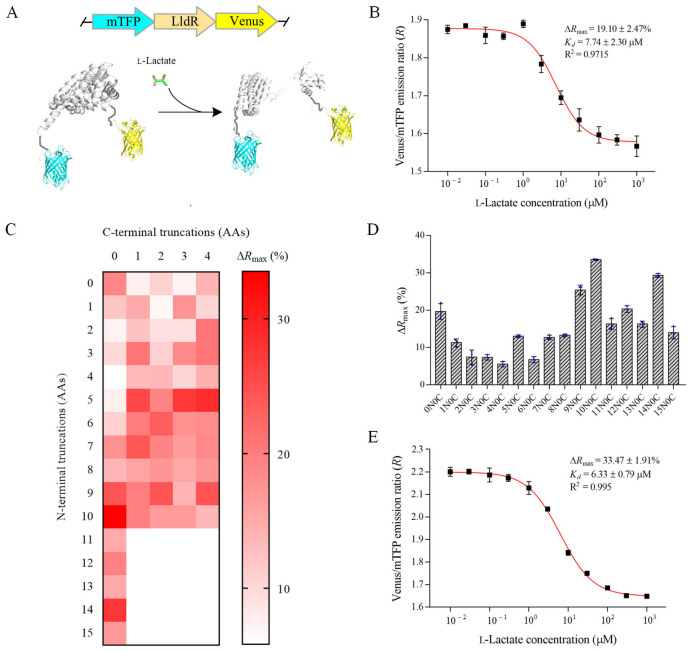
Design and optimization of FILLac. (**A**) Schematic representation of the predicted conformational changes of the l-lactate biosensor FILLac in the presence or absence of l-lactate. The structure of *ST*LldR shown was predicted based on its amino acid sequence, and the structures of mTFP and Venus shown were downloaded from PDB (PDB ID: 4R6D for mTFP and 3AKO for Venus). (**B**) Dose-response curve of FILLac_0N0C_ for increasing concentration (10 nM to 1 mM) of l-lactate. The fluorescence emission ratio (Venus to mTFP) of FILLac_0N0C_ decreased after l-lactate binding. (**C**) Heat map of variants with N-terminal or C-terminal amino acid truncations of *ST*LldR to Δ*R*_max_. The color indicates the value of Δ*R*_max_, and white indicates undetected variants. (**D**) Comparison of the Δ*R*_max_ of biosensor variants based on the N-terminal amino acid truncation of *ST*LldR. (**E**) Dose-response curve of the optimized variant FILLac_10N0C_ for increasing concentration (10 nM to 1 mM) of l-lactate. All data shown are mean ± s.d. (*n* = 3 independent experiments).

FRET is an energy transfer process in which the donor fluorophore in the electronic excited state transfers energy to the acceptor fluorophore through resonant coupling [25]. FRET-based biosensors, which are composed of a sensing domain and two fluorophores, allow quantification of metabolites based on the ligand-binding induced changes in distance and/or orientation of two fluorophores and FRET efficiency [25]. In this study, a fluorescent l-lactate biosensor was constructed based on *ST*LldR and FRET (Figure 2A). The optimized cyan and yellow fluorescent proteins, mTFP and Venus, were fused to the N-terminus and C-terminus of *ST*LldR, respectively (Figure S2, Supplementary Material). The constructed fluorescent l-lactate biosensor was named as FILLac_0N0C_ and expressed in *E. coli* BL21(DE3). FILLac_0N0C_ exhibited l-lactate-dependent increases in the emission peak at 492 nm and decrease in the emission peak at 526 nm with excitation at 430 nm (Figure S3, Supplementary Material). Thus, the structural change of *ST*LldR after l-lactate binding may lead to an unfavorable orientation and/or extended distance of mTFP and Venus, resulting in the decrease in FRET. In addition, l-lactate decreased the fluorescence emission ratio of FILLac_0N0C_ in a dose-dependent manner with a maximum emission ratio change (Δ*R*_max_) of 19.10 ± 2.47% and an apparent dissociation constant (*K_d_*) of 7.74 ± 2.30 μM (Figure 2B).

Then, the biosensor was optimized for its magnitude of response to l-lactate. Sixty truncation variants were constructed by truncating the N- and C-terminal amino acids of *ST*LldR (Figure 2C). As shown in Figure 2D, truncation of N-terminal amino acids of *ST*LldR may significantly increase the Δ*R*_max_ of the sensor. The variant with a ten amino acids truncation of N-terminal of *ST*LldR, designated as FILLac_10N0C_, showed the largest change in fluorescence intensity (Δ*R*_max_ = 33.47 ± 1.91%) and a *K_d_* of 6.33 ± 0.79 μM (Figure 2E). The limits of detection (LOD) of FILLac_10N0C_ for l-lactate was 0.68 μM, and the linear detection range was 0.76−51.79 μM. Artificial linkers including flexible linker G_4_ and rigid linker KL were further added between the truncated *ST*LldR and two fluorescent proteins. However, none of the six constructed variants exhibited a larger Δ*R*_max_ than that of FILLac_10N0C_ (Figure S4, Supplementary Material). Therefore, FILLac_10N0C_ was selected as the optimal l-lactate biosensor for intensive study in subsequent experiments.

### 3.3. Characterization of the Optimal l-Lactate Biosensor FILLac_10N0C_

**Figure 3:**
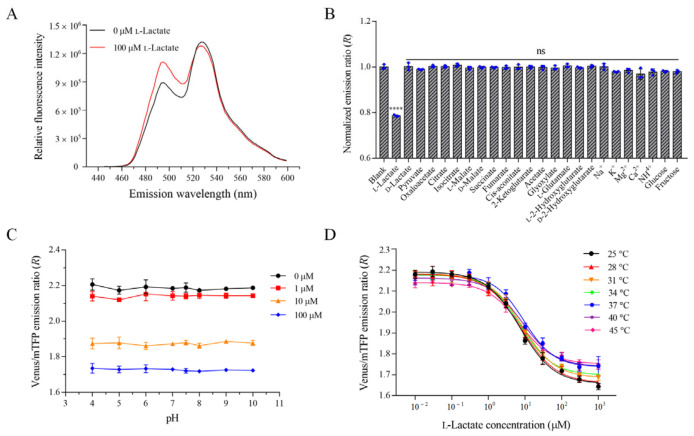
*In-vitro* characterization of FILLac_10N0C_. (**A**) Fluorescence emission spectra of FILLac_10N0C_ in the absence of l-lactate (black) and in the presence of 100 μM l-lactate (red) with excitation at 430 nm. (**B**) Specificity analysis of FILLac_10N0C_. The fluorescence emission ratio changes of FILLac_10N0C_ were determined in the presence of 50 μM l-lactate, d-lactate, pyruvate, oxaloacetate, acetate, glyoxylate, citrate, isocitrate, d-malate, l-malate, succinate, fumarate, cis-aconitate, 2-ketoglutarate, d-2-hydroxyglutarate, l-2-hydroxyglutarate, l-glutamate, Na^+^, K^+^, Ca^2+^, Mg^2+^, NH_4_^+^, glucose, and fructose. (**C**) pH stability analysis of FILLac_10N0C_. Fluorescence emission ratios of FILLac_10N0C_ in the presence of l-lactate (0, 1, 10, and 100 μM) were determined at different pH values. (**D**) Temperature stability analysis of FILLac_10N0C_. Dose-response curves of FILLac_10N0C_ for increasing concentration (10 nM to 1 mM) of l-lactate were determined at different temperatures, ranging from 25 °C to 45 °C. All data shown are mean ± s.d. (*n* = 3 independent experiments). The significance of the data was analyzed by a two-tailed, unpaired *t*-test; ****, *p* < 0.0001; ns, no significant difference (*p* ≥ 0.05).

The properties of the optimal variant FILLac_10N0C_ were further characterized. As shown in Figure 3A, FILLac_10N0C_ also exhibited an l-lactate-dependent increase in the emission peak at 492 nm and a decrease in the emission peak at 526 nm. The increase in the emission peak at 492 nm was more significant than that of FILLac_0N0C_ (Figure 3A and Figure S3A, Supplementary Material). Only l-lactate significantly reduced the fluorescence emission ratio of FILLac_10N0C_. d-Lactate, pyruvate, oxaloacetate, acetate, glyoxylate, citrate, isocitrate, d-malate, l-malate, succinate, fumarate, cis-aconitate, 2-ketoglutarate, d-2-hydroxyglutarate, l-2-hydroxyglutarate, l-glutamate, Na^+^, K^+^, Ca^2+^, Mg^2+^, NH_4_^+^, glucose and fructose did not change the emission ratio (Figure 3B). Detection of l-lactate by FILLac_10N0C_ was not affected in the presence of 50 μM d-lactate or pyruvate (Figure S5B–E, Supplementary Material). The pH sensitivity of FILLac_10N0C_ was determined. There were no detectable changes in the Venus to mTFP ratio in the pH range from 4.0 to 10.0 (Figure 3C). The dose-response curves of FILLac_10N0C_ for l-lactate were also determined at different temperatures (Figure 3D). The results showed that the affinity of FILLac_10N0C_ to l-lactate was unaffected under the test temperatures (Figure S5F, Supplementary Material).

### 3.4. Performance of FILLac_10N0C_ in l-Lactate Quantitation

**Figure 4:**
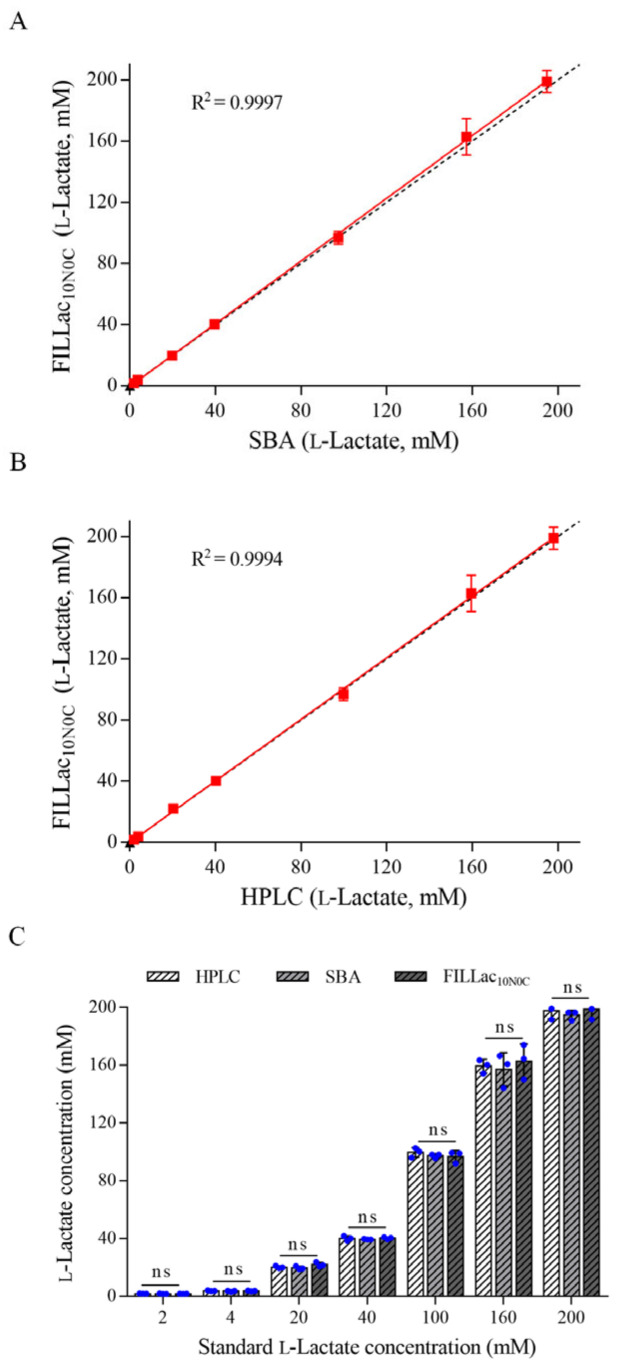
Performance of FILLac_10N0C_ in l-lactate quantitation. (**A**) Comparison of the quantitative analysis of l-lactate by using HPLC and FILLac_10N0C_. Concentrations detected by HPLC were used as control (*x*-axis). The black dashed line is a reference line with a slope of 1. (**B**) Comparison of the quantitative analysis of l-lactate by using SBA-40D bioanalyzer and FILLac_10N0C_. Concentrations detected by SBA-40D bioanalyzer were used as control (*x*-axis). The black dashed line is a reference line with a slope of 1. (**C**) Quantification of l-lactate by HPLC, SBA-40D bioanalyzer and FILLac_10N0C_. Standard concentrations of l-lactate are 2, 4, 20, 40, 100, 160, and 200 mM. All data shown are mean ± s.d. (n = 3 independent experiments). The significance of the data was analyzed by a two-tailed, unpaired *t*-test; ns, no significant difference (*p* ≥ 0.05).

Different concentrations of l-lactate were added to the fermentation medium to simulate the samples for l-lactate detection. l-Lactate concentrations in these samples were detected by FILLac_10N0C_, SBA-40D bioanalyzer, and HPLC, respectively. As shown in Figure 4A,B, the results of l-lactate detection by FILLac_10N0C_ showed high agreement (R^2^ > 0.999) with those of HPLC and SBA-40D bioanalyzer. There was no significant difference in the detection results of the three methods (Figure 4C). The accuracy and precision of FILLac_10N0C_, SBA-40D bioanalyzer, and HPLC for quantitative detection of l-lactate were also analyzed. The results showed that the developed biosensor FILLac_10N0C_ has high accuracy and precision for the quantitative detection of l-lactate (Table S2, Supplementary Material).

### 3.5. Quantitation of l-Lactate in Different Fermentation Samples by FILLac_10N0C_

**Figure 5:**
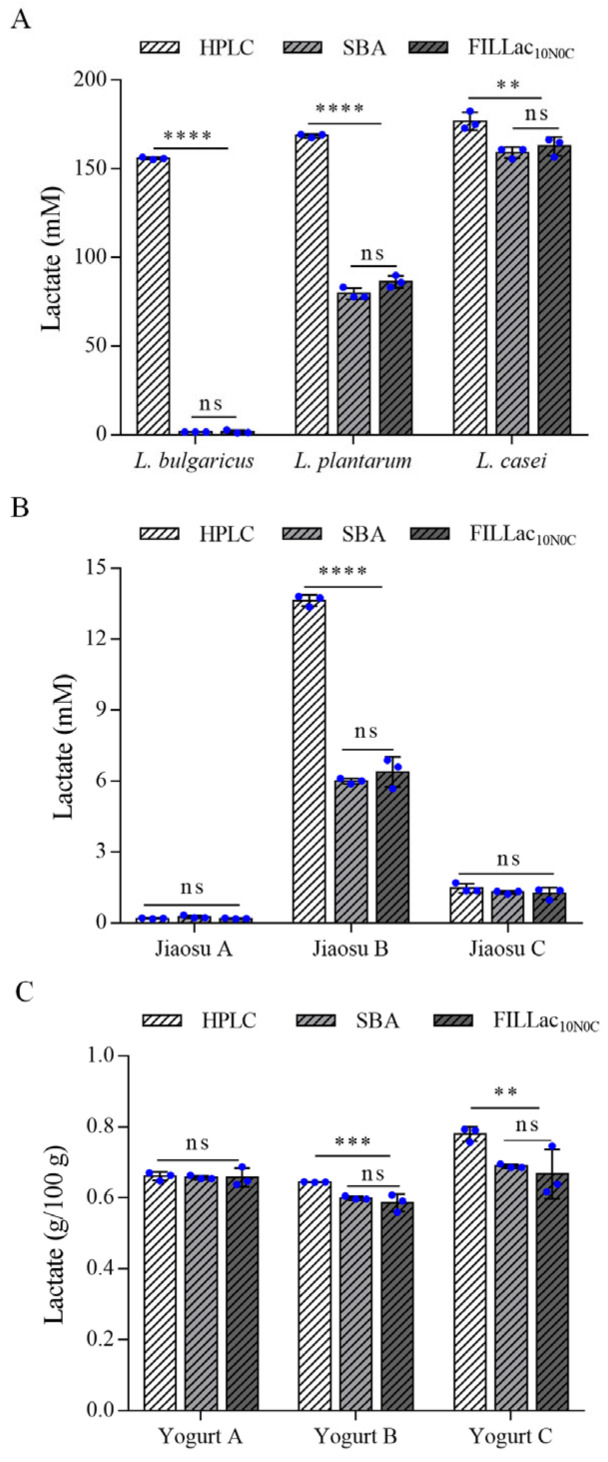
Quantification of l-lactate in various samples using the sensor FILLac_10N0C_. (**A**) Comparison of the quantification results of lactate in fermentation samples produced by *L. bulgaricus* ATCC 11842, *L. plantarum* ATCC 14917 and *L. casei* ATCC 334 by HPLC, SBA-40D bioanalyzer, and FILLac_10N0C_. (**B**) Comparison of the quantification results of lactate in three Jiaosu samples by HPLC, SBA-40D bioanalyzer and FILLac_10N0C_. (**C**) Comparison of the quantification results of lactate in three yogurt samples by HPLC, SBA-40D bioanalyzer and FILLac_10N0C_. All data shown are mean ± s.d. (*n* = 3 independent experiments). The significance of the data was analyzed by a two-tailed, unpaired *t*-test; **, *p* < 0.01; ***, *p* < 0.001; ****, *p* < 0.0001; ns, no significant difference (*p* ≥ 0.05).

Nowadays, lactate is mainly produced by microbial fermentation [40,41] and *Lactobacillus* is a common genus for lactate production [42]. Three *Lactobacillus* strains, *L. casei* ATCC 334, *L. plantarum* ATCC 14917 and *L. bulgaricus* ATCC 11842 were reported to mainly produce l-lactate [43], d,l-lactate [44], and d-lactate [41], respectively. To identify the feasibility of the sensor FILLac_10N0C_ in quantification of l-lactate in bacterial fermentation samples, these three *Lactobacillus* strains were used for fermentative production of lactate. Then, the concentrations of lactate in the fermentation samples were detected by FILLac_10N0C_, SBA-40D bioanalyzer, and HPLC, respectively. Chiral chromatographic analysis revealed the presence of two chiral isomers of lactate in all three fermentation samples (Figure S6, Supplementary Material). SBA-40D bioanalyzer is a commercialized electrochemical biosensor for l-lactate detection. It uses immobilized l-lactate oxidase (l-LOx) as its biological recognition element for selective detection of l-lactate [32]. Generally consistent with expectations, SBA-40D bioanalyzer could not detect d-lactate in the three samples and the results of SBA-40D bioanalyzer were lower than those of HPLC (Figure 5A). Importantly, the results of FILLac_10N0C_ and SBA-40D bioanalyzer were consistent with no significant difference (Figure 5A). Thus, FILLac_10N0C_, like the commercial SBA-40D bioanalyzer, can be used for the selective detection of l-lactate in fermentation samples.

### 3.6. Determination of l-Lactate in Food Samples by FILLac_10N0C_

l-Lactate is present in various fermented foods and can be used as an indicator for food flavor and quality [45,46]. The application of FILLac_10N0C_ in detection of l-lactate in fermented foods such as yogurt and Jiaosu was also analyzed. As shown in Figure 5B,C, the results of FILLac_10N0C_ and SBA-40D bioanalyzer with all of the three commercial yogurt and Jiaosu were identical to each other. Due to the presence of d-lactate in Jiaosu B, Yogurt B, and Yogurt C (Figure S7, Supplementary Material), the results of HPLC with these samples were higher than those of FILLac_10N0C_ and SBA-40D bioanalyzer. Preliminary spiking experiments on three actual Jiaosu samples using FILLac_10N0C_ were also performed and satisfactory recoveries of 93.15–97.47% were obtained (Table S3, Supplementary Material).

**Figure 6:**
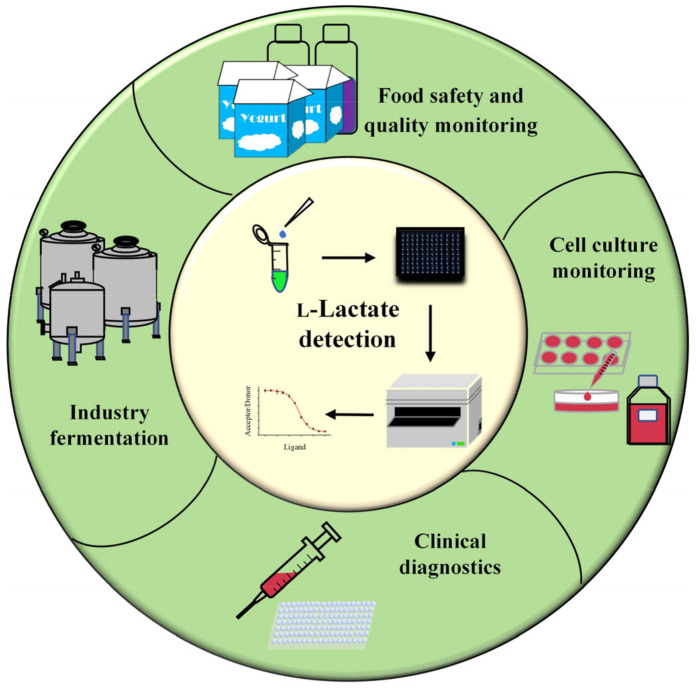
Schematic of the detection and application of FILLac_10N0C_. The l-lactate concentration in different biological samples can be quantified by mixing the FILLac_10N0C_ with microvolume samples in black 96-well microplates and detected by a fluorescence microplate detector. The FILLac_10N0C_ may be widely used in the fields of food safety and quality control, industry fermentation, clinical diagnostics, and cell culture processes.

Selective l-lactate detection is ultimately important for food, clinical, and fermentation applications [19,45,46]. In this study, a selective fluorescent biosensor, FILLac_10N0C_, was constructed for l-lactate detection. Compared with other reported fluorescent lactate biosensors, a unique feature of FILLac_10N0C_ is that it uses *ST*LldR, an l-lactate selective aTF, as its recognition element. Exposure of FILLac_10N0C_ to d-lactate did not change fluorescence emission ratio significantly, with a high *K_d_* of 287.6 ± 66.5 μM (Figure S8, Supplementary Material). The commercial d-lactate we used is 98% enantiopure. The observed response of FILLac_10N0C_ to d-lactate at high concentrations was likely a reflection of this impurity. The results of FILLac_10N0C_ with bacterial fermentation and food samples showed high agreement (R^2^ > 0.999) with that of SBA-40D bioanalyzer, a commercial biosensor for l-lactate detection. Compared with the SBA-40D bioanalyzer, FILLac_10N0C_ possesses a distinctive advantage of compatible with 96- or 384-well plates for high-throughput l-lactate detection. After stored at –80 ℃ for six months, FILLac_10N0C_ exhibited a Δ*R*_max_ of 32.86 ± 2.33% and a *K_d_* of 5.85 ± 0.92 μM (Figure S9, Supplementary Material), which were in good agreement with those of the freshly prepared biosensor (Δ*R*_max_ of 33.47 ± 1.91% and *K_d_* of 6.33 ± 0.79 μM). Due to the good performance of FILLac_10N0C_ in l-lactate detection, it may be a promising choice for the selective monitoring of extracellular l-lactate concentration in various biological samples (Figure 6).

l-Lactate is a crucial metabolite with diverse metabolic and signaling roles [47]. Some fluorescent sensors such as Laconic, eLACCO1.1, LiLac, etc., have been established for monitoring intracellular lactate metabolism [26,29,31]. The lactate produced in human is mainly l-isomer and thus the stereoselectivity of these sensors received insufficient attention. However, human can acquire exogenously d-lactate in foods, catabolize chemicals such as propylene glycol into d-lactate, and uptake d-lactate produced by intestinal bacteria [48]. For example, patients with short bowel syndrome may suffer from a complication called d-lactic acidosis, and d-lactate levels in plasma may be higher than 3 mM [49]. d-Lactate can also be endogenously generated in various types of cancer by methylglyoxal metabolism [48,50]. In addition, d-lactate exists in various model species such as *Arabidopsis thaliana* [51] and *Saccharomyces cerevisiae* [52]. The presence of d-lactate under these cases may interfere with the detection of l-lactate by unselective biosensors. Considering the recognitional capacity of *ST*LldR toward l-lactate, *ST*LldR is an ideal recognition element for construction of intracellular l-lactate biosensors with stereoselectivity. FILLac_10N0C_ now exhibited good performance in monitoring extracellular l-lactate concentration. However, it has a rather small dynamic range and an inappropriate high affinity to l-lactate, which limit its application in detecting lactate fluctuations *in-vivo*. A more responsive derivative of FILLac_10N0C_ with much higher magnitude of response and an appropriate affinity is need for monitoring l-lactate concentrations in living cells.

## 4. Conclusions

In this study, *ST*LldR, the regulator of lactate utilization operon in *S. Typhimurium* LT2, was identified to specifically sense l-lactate. A stereoselective l-lactate biosensor was then constructed and systematically optimized. The quantitation results of l-lactate in bacterial fermentation samples, Jiaosu and yogurt using the optimal biosensor FILLac_10N0C_ showed high agreement with that of the commercial SBA-40D bioanalyzer. With its desirable properties such as high sensitivity, specificity, and compatibility with high-throughput detection, l-lactate biosensor FILLac_10N0C_ may be a promising tool in quantitation of l-lactate in various biological samples.

## Data Availability

Not applicable.

## Supplementary Materials

The following supporting information can be downloaded at: https://www.mdpi.com/article/10.3390/bios12121111/s1. Figure S1: SDS-PAGE analysis of purified *Ec*LldR (A), *Pa*LldR (B), *Pf*LldR (C), and *ST*LldR (D). Figure S2: Amino acid sequences of FILLac_0N0C_. Figure S3: Spectral properties of FILLac_0N0C_. Figure S4: Heat map of FILLac_10N0C_ variants with different artificial linkers added between *ST*LldR and fluorescent proteins to Δ*R*_max_. Figure S5: In vitro characterization of FILLac_10N0C_. Figure S6: HPLC analysis of the chirality of lactate in different fermentation samples. Figure S7: HPLC analysis of the chirality of lactate in Jiaosu and yogurt samples. Figure S8: Dose-response curve of FILLac_10N0C_ for increasing concentrations (100 nM to 10 mM) of d-lactate. Figure S9: Dose-response curve of FILLac_10N0C_ for increasing concentrations (100 nM to 10 mM) of l-lactate after stored at –80 ℃ for six months. Table S1: Strains and plasmids used in this study. Table S2: Evaluation of the performance of FILLac_10N0C_ for quantification of l-lactate in various biological samples. Table S3: Evaluation of the accuracy of FILLac_10N0C_ for quantification of l-lactate in biological samples.
