# Supplementary material for: A Selective Fluorescent l-Lactate Biosensor Based on an l-Lactate-Specific Transcription Regulator and Förster Resonance Energy Transfer
Source: Biosensors (Basel). 2022 Dec 1;12(12):1111. doi: 10.3390/bios12121111 (PMC9775004; doi:10.3390/bios12121111)
Supplement: Supplementary file 1 [file biosensors-12-01111-s001.zip › biosensors-2042861-supplementary.pdf]

Supplementary Material

A Selective Fluorescent L-Lactate Biosensor Based on an L-Lactate-Specific Transcription Regulator and Förster Resonance Energy Transfer

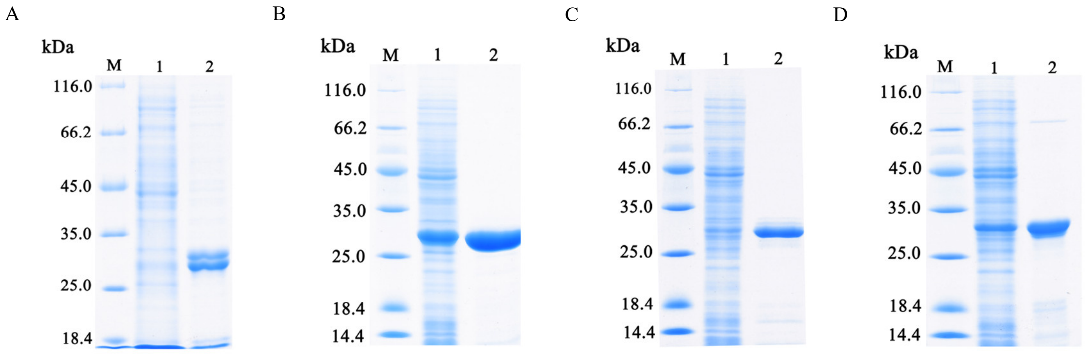

Figure S1. SDS-PAGE analysis of purified *Ec*LldR (**A**), *Pa*LldR (**B**), *Pf*LldR (**C**), and *ST*LldR (**D**). Lane M, molecular weight markers; lane 1, crude extract of *Escherichia coli* BL21(DE3) harboring expressing plasmids of different LldRs; lane 2, different purified His$_6$-tagged LldRs using a HisTrap column.

MVSKGEETTMGVIKPDMKIKLKMEGNVNGHAFVIEG
EGEGKPYDGTNTINLEVKEGAPLPFSYDILTTAFAYGN
RAFTKYPDDIPNYFKQSFPEGYSWERTMTFEDKGIVKV
KSDISMEEDSFIYEIHLKGENFPPNGPVMQKKTTGWDA
STERMYVRDGVLKGDVKHKLLLEGGGHHRVDFKTIY
RAKKAVKLPDYHFVDHRIEILNHDKDYNKVTVYESAV
ARNSTDGMDELYKELMIVMPKRLSDEIASRVRALIEEQ
NLEAGMKLPAERQLALQLGVSRNSLREALAKLVSEGV
LVSRRGGGTFVRWQHETWSEQNIVQPLKMLMANDPD
YSFDILEARHAIEASTAWHAAMRATAADKEKIRLCFDA
TLSEDPDLASQADVRFHLAIAEASHNVVLLQTMRGFF
DVLQSSVKQSRQRMYLVPPVFSKLTEQHQAVMDAILD
GNAEGARKAMMAHLSFVHTTIKRFDEDQARQARITRL
PGDHNEMTRENKSVDMVSKGEELFTGVVPILVELDGD
VNGHKFSVSGEGEGDATYGKLTLKLICTTGKLPVPWP
TLVTTLGYGLQCFARYPDHMKQHDFFKSAMPEGYVQ
ERTIFFKDDGNYKTRAEVKFEGDTLVNRIELKGIDFKE
DGNILGHKLEYNYNSHNVYITADKQKNGIKANFKIRH
NIEDGGVQLADHYQQNTPIGDGPVLLPDNHYLSYQSA
LSKDPNEKRDHMVLLEFVTAAGITLGMDELYK*

Figure S2. Amino acid sequences of FILLac$_{0N0C}$. The sequences of mTFP, *ST*LldR and Venus are highlighted in cyan, grey and yellow, respectively. EL and VD are the amino acid sequences of the restriction sites SacI and SalI, respectively.

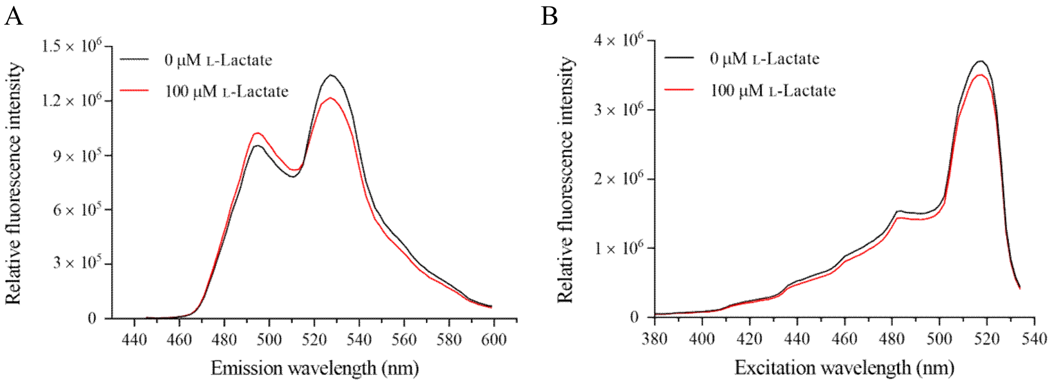

Figure S3. Spectral properties of FILLac$_{0N0C}$. (**A**) Fluorescence emission spectrum changes of 1 μM FILLac$_{0N0C}$ with (red) or without (black) 100 μM L-lactate were indicated, excitation was measured at 430 nm with emission from 445 nm to 600 nm in steps of 2 nm. (**B**) Fluorescence excitation spectrum changes of 1 μM FILLac$_{0N0C}$ with (red) or without (black) 100 μM L-lactate were indicated, emission was measured at 550 nm with excitation from 380 nm to 535 nm in steps of 2 nm.

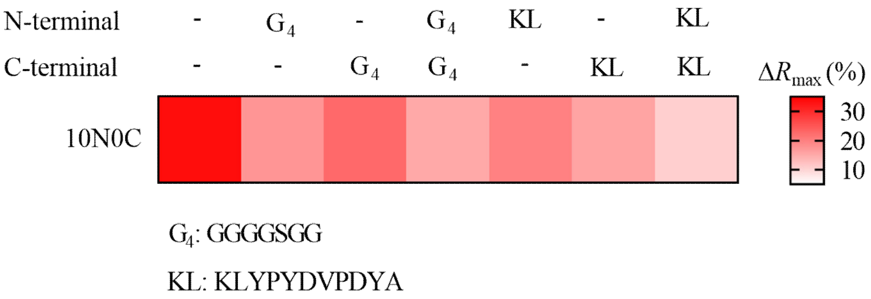

Figure S4. Heat map of FILLac$_{10N0C}$ variants with different artificial linkers added between *ST*LldR and fluorescent proteins to ΔR_{max}. G4 and KL are the flexible linker Gly-Gly-Gly-Gly-Ser-Gly-Gly and the rigid linker Lys-Leu-Tyr-Pro-Tyr-Asp-Val-Pro-Asp-Tyr-Ala, respectively.

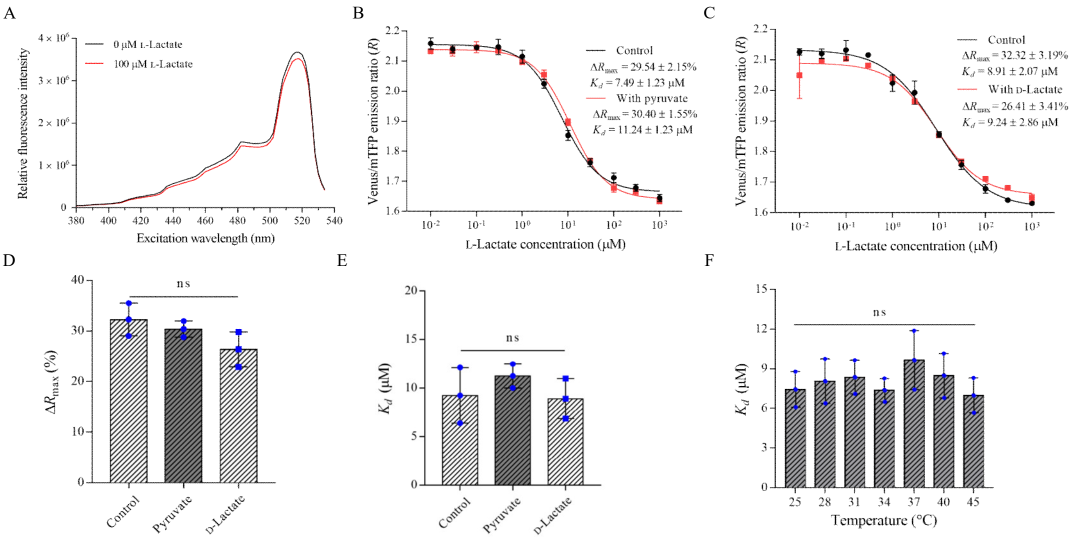

Figure S5. *In vitro* characterization of FILLac$_{10N0C}$. (**A**) Fluorescence excitation spectrum changes of 1 μM FILLac$_{10N0C}$ with (red) or without (black) 100 μM L-lactate were indicated, emission was measured at 550 nm with excitation from 380 nm to 535 nm in 2 nm steps. (**B**) Dose-response curves of

FILLac$_{10N0C}$ for L-lactate in the presence (red) or absence (black) of 50 μM pyruvate. (**C**) Dose-response curves of FILLac$_{10N0C}$ for L-lactate in the presence (red) or absence (black) of 50 μM D-lactate. (**D**) Comparison of the ΔR_{max} of FILLac$_{10N0C}$ in the presence of 50 μM D-lactate or pyruvate. (**E**) Comparison of the K_d values of FILLac$_{10N0C}$ in the presence of 50 μM D-lactate or pyruvate. (**F**) K_d values of FILLac$_{10N0C}$ for L-lactate determined at different temperatures. All data shown are means ± s.d. (n = 3 independent experiments). The significance of the data was analyzed by a two-tailed, unpaired t-test; ns, no significant difference ($p \geq 0.05$).

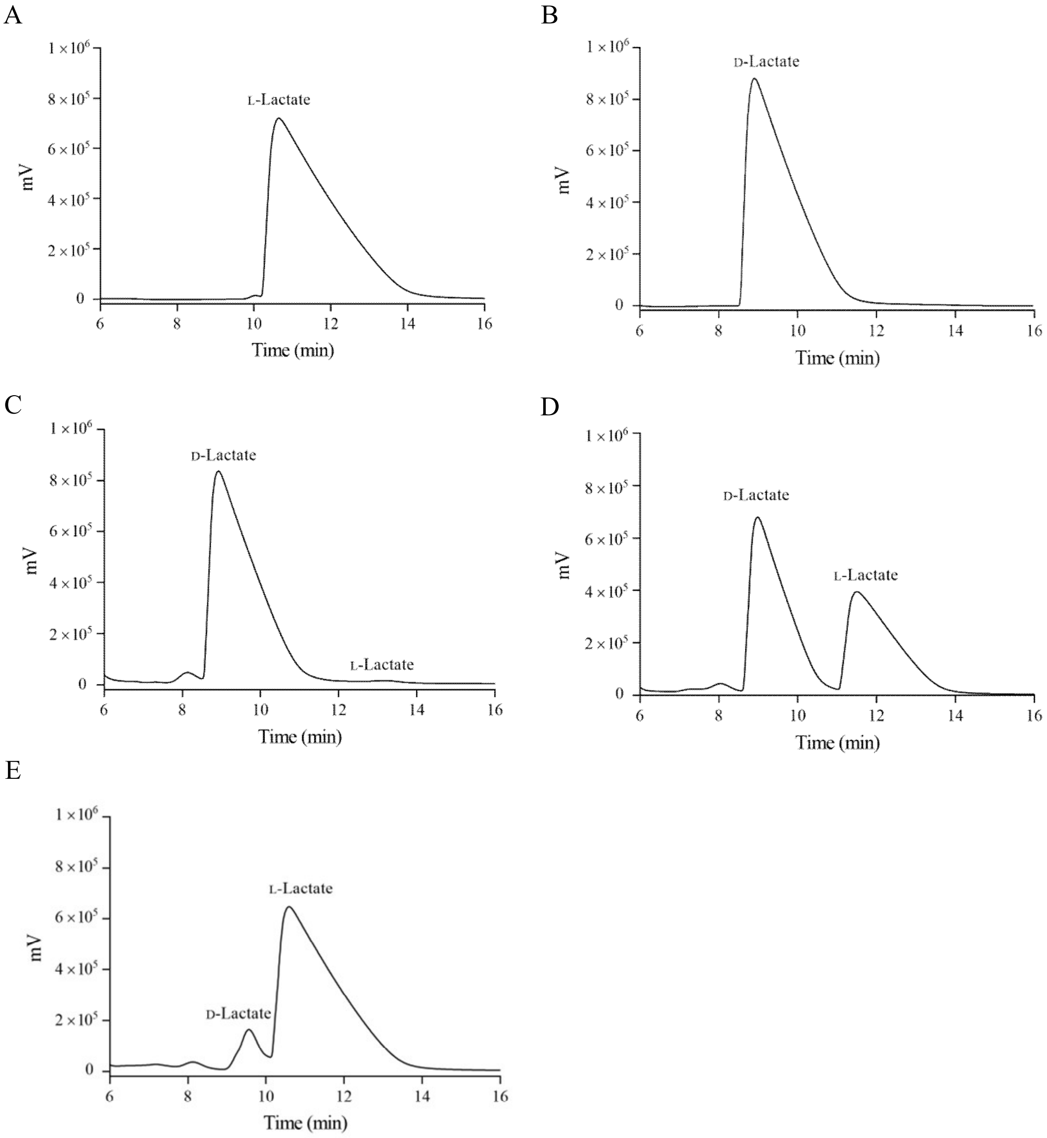

Figure S6. HPLC analysis of the chirality of lactate in different fermentation samples. (**A**,**B**) HPLC analysis of L-lactate standard (**A**) and D-lactate standard (**B**) by using a chiral column. (**C**–**E**) HPLC analysis of the chirality of lactate in fermentation samples produced by *L. bulgaricus* ATCC 11842 (**C**), *L. plantarum* ATCC 14917 (**D**), and *L. casei* ATCC 334 (**E**) by using a chiral column.

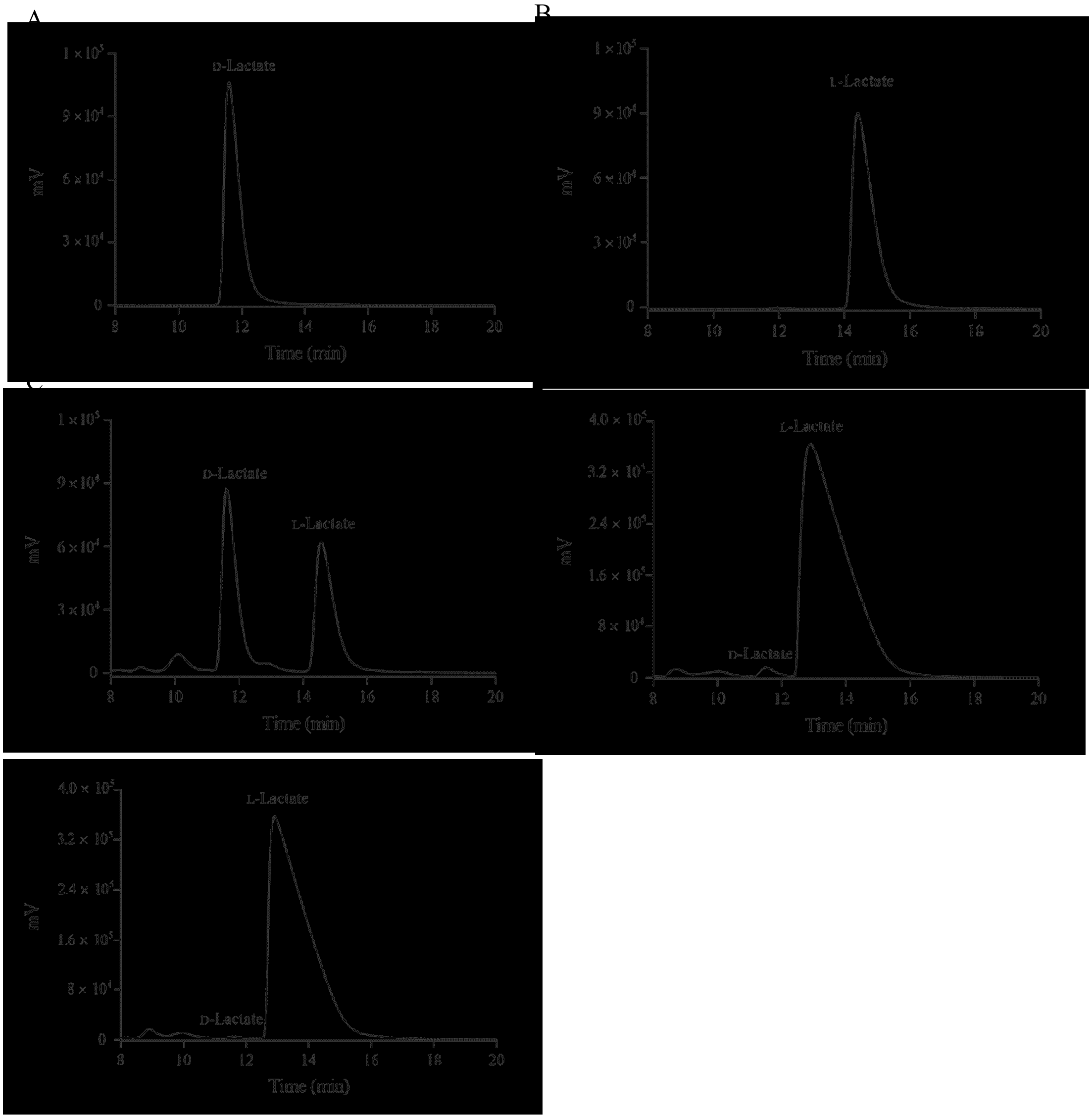

Figure S7. HPLC analysis of the chirality of lactate in Jiaosu and yogurt samples. (**A**,**B**) HPLC analysis of L-lactate standard (**A**) and D-lactate standard (**B**) by using a chiral column. (**C**–**E**) HPLC analysis for Jiaosu B (**C**), Yogurt B (**D**) and Yogurt C (**E**) by using a chiral column.

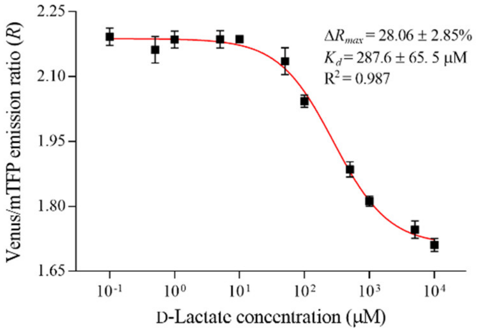

Figure S8. Dose-response curve of FILLac$_{10N0C}$ for increasing concentrations (100 nM to 10 mM) of D-lactate.

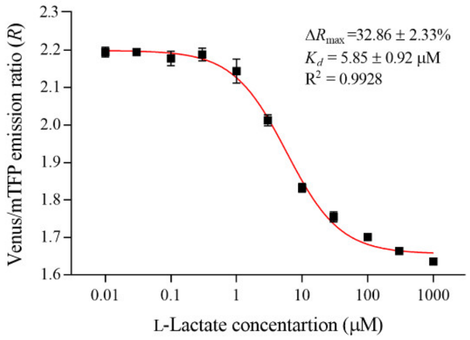

Figure S9. Dose-response curve of FILLac10N0C for increasing concentrations (100 nM to 10 mM) of L-lactate after stored at −80°C for six months.

Table S1. Strains and plasmids used in this study.

Strain and Plasmid	Relevant Characteristics [a]	Source and Reference
**Strain**		
E. coli BL21(DE3)	F⁻ *ompT hsdSB* (*rB- mB-*) *gal* (λ c *I* 857 *ind1 Sam7 nin*5 l*ac*UV5-T7*gene*1) *dcm* (DE3)	Invitrogen
E. coli BL21(DE3) (pET28a-*lldR*)	*E. coli* BL21(DE3) harboring the plasmid pET28a-*lldR*	This study
E. coli BL21(DE3) (pETDuet-*mTFP-lldR-Venus*)	*E. coli* BL21(DE3) harboring the plasmid pETDuet- *mTFP-lldR-Venus*	This study
Lactobacillus bulgaricus ATCC 11842	Wild-type D-lactate producing strain	ATCC [b]
L. plantarum ATCC 14917	Wild-type D, L-lactate producing strain	ATCC [b]
L. casei ATCC 334	Wild-type L-lactate producing strain	ATCC [b]
**Plasmid**		
pET28a	Expression vector; Km^r	Novagen
pET28a-*EclldR*	pET28a contained *lldR* of *E. coli* MG1655; Km^r	This study
pET28a-*PalldR*	pET28a contained *lldR* of *Pseudomonas aeruginosa* PAO1; Km^r	(Xiao et al., 2022)
pET28a-*PflldR*	pET28a contained *lldR* of *P. fluorescens* A506; Km^r	This study
pET28a-*STlldR*	pET28a contained *lldR* of *Salmonella enterica serovar Typhimurium* LT2; Km^r	This study
pETDuet-1	Expression vector; Amp^r	Novagen
pETDuet- *mTFP-Venus*	pETDuet-1 contained the genes of *mTFP* and *Venus*; Amp^r	(Kang et al., 2021)

[a] Km^r, kanamycin resistant; Amp^r, ampicillin resistant. [b] ATCC: American Type Culture Collection.

Table S2. Evaluation of the performance of FILLac10N0C for quantification of L-lactate in various biological samples.

Approach	Concentration (mM)				Accuracy (%) [a]				Precision (RSD%) [b]
Standard	Sample 1	Sample 2	Sample 3	Sample 4	Sample 1	Sample 2	Sample 3	Sample 4	
	2	4	20	40	2	4	20	40	
HPLC	1.97 ± 0.11	3.98 ± 0.17	20.30 ± 0.75	40.29 ± 1.35	98.67	99.67	101.52	100.72	1.07
SBA	1.89 ± 0.08	3.70 ± 0.09	19.79 ± 0.94	39.59 ± 0.26	94.50	92.50	98.95	98.98	2.82
FILLac10N0C	1.90 ± 0.07	3.86 ± 0.19	22.08 ± 1.28	40.42 ± 0.59	95.00	96.50	110.43	101.04	5.97

[a] $\text{Accuracy\%} = \frac{\text{Concentration determined by different methods}}{\text{Defined concentration}}$. [b] $\text{Precision\%} = \frac{\text{Standard derivation of accuracy}}{\text{Mean value of accuracy}}$.

Table S3. Evaluation of the accuracy of FILLac10N0C for quantification of L-lactate in biological samples.

Sample	Concentration (mM)			Recovery (%) [d]
	**Actual Sample** [a]	**Added** [b]	**Spiked Sample** [c]	
Jiaosu 1	0.36 ± 0.03	5.00	5.23 ± 0.24	97.47
Jiaosu 2	8.83 ± 0.27	5.00	13.11 ± 1.43	93.15
Jiaosu 3	1.53 ± 0.12	5.00	6.05 ± 0.32	96.80

[a] L-Lactate concentrations measured in Jiaosu samples using FILLac10N0C. [b] Added extra 5 mM L-lactate to Jiaosu samples. [c] L-Lactate concentrations measured in spiked samples using FILLac10N0C. [d] Recovery% = $\frac{\text{Concentration of L-lactate in spiked sample} - \text{Concentration of L-lactate in original actual sample}}{\text{Concentration of the added L-lactate}}$ ×100%.

References

1. Kang, Z.Q.; Zhang, M.M.; Gao, K.Y.; Zhang, W.; Meng, W.S.; Liu, Y.D.; Xiao, D.; Guo, S.T.; Ma, C.Q.; Gao, C.; et al. An L-2-hydroxyglutarate biosensor based on specific transcriptional regulator LhgR. *Nat. Commun.* **2021**, *12*, 3619. http://doi.org/10.1038/s41467-021-23723-7.
2. Xiao, D.; Hu, C.; Xu, X.; Lü, C.; Wang, Q.; Zhang, W.; Gao, C.; Xu, P.; Wang, X.; Ma, C. A D,L-lactate biosensor based on allosteric transcription factor LldR and amplified luminescent proximity homogeneous assay. *Biosens. Bioelectron.* **2022**, *211*, 114378. https://doi.org/10.1016/j.bios.2022.114378.
